# Urban air quality forecasting based on multi-dimensional collaborative Support Vector Regression (SVR): A case study of Beijing-Tianjin-Shijiazhuang

**DOI:** 10.1371/journal.pone.0179763

**Published:** 2017-07-14

**Authors:** Bing-Chun Liu, Arihant Binaykia, Pei-Chann Chang, Manoj Kumar Tiwari, Cheng-Chin Tsao

**Affiliations:** 1 Research Institute of Circular Economy, Tianjin University of Technology, Tianjin, P.R. China; 2 Department of Industrial and Systems Engineering, Indian Institute of Technology Kharagpur, Kharagpur, India; 3 Department of Information Management, Yuan Ze University, Taoyuan, Taiwan, ROC; 4 Office of Academic Affairs, Zhuhai College of Beijing Institute of Technology, Zhuhai, China; National Sun Yat-sen University, TAIWAN

## Abstract

Today, China is facing a very serious issue of Air Pollution due to its dreadful impact on the human health as well as the environment. The urban cities in China are the most affected due to their rapid industrial and economic growth. Therefore, it is of extreme importance to come up with new, better and more reliable forecasting models to accurately predict the air quality. This paper selected Beijing, Tianjin and Shijiazhuang as three cities from the Jingjinji Region for the study to come up with a new model of collaborative forecasting using Support Vector Regression (SVR) for Urban Air Quality Index (AQI) prediction in China. The present study is aimed to improve the forecasting results by minimizing the prediction error of present machine learning algorithms by taking into account multiple city multi-dimensional air quality information and weather conditions as input. The results show that there is a decrease in MAPE in case of multiple city multi-dimensional regression when there is a strong interaction and correlation of the air quality characteristic attributes with AQI. Also, the geographical location is found to play a significant role in Beijing, Tianjin and Shijiazhuang AQI prediction.

## Introduction

Air quality has a huge impact on the quality of living, the well-being of the population as well as the image of the city. With the increase in population in urban areas, there has been an increase in the development of the industries as well as the consumption of fossil fuels. This has led to the increase in air pollution in China [1]. The dreadful effects of air quality in the capital city of China—Beijing due to increase in population which has led to increase in number of vehicles as well as increase in fuel consumption has been the point of discussion for many researchers [2]. It can be said that the progress of the human society is at the expense of our lives as well as the environment [3]. The goal of environment sustainability is difficult to achieve due to excessive air pollution in urban cities of China including Beijing, Tianjin and Shijiazhuang [4]. Airborne particulate matter (PM) is especially detrimental to health and has been estimated to cause between 3 and 7 million deaths every year, primarily causing cardiorespiratory disease [5]. NO and NO_2_ are the two air pollutants which also cause respiratory diseases proven by using multiple linear regression to analysis the correlation coefficient between the outpatient visits and air pollution [6].

Beijing-Tianjin-Hebei economic zone is the capital of Northern China’s largest urban agglomeration that has witnessed rapid economic and population growth. Shijiazhuang is the capital and largest city of North China's Hebei Province. Due to the rapid economic and population growth it has encountered a series of environment protection and sustainable development related issues. In particular air pollution has a direct impact on the health of the residents as well as the quality of living and image of the city. The region faces industrial emissions, large scale urban construction and other characteristics of a fast growing region which are dangerous environmental threats of air pollution to the public.

Air Quality Index (AQI) is a widely used index for public understanding and for evaluation of air pollution on human health indicators [7]. In 1972, the US Environmental Protection Agency (EPA) first proposed the Pollution Standard Index (PSI). In 1999, EPA proposed the Air Quality Index (AQI) which is now used worldwide. In China, the AQI is measured through real time monitoring of Air Quality Data obtained through the conversion process which is very important for future AQI forecast. AQI forecast research is currently focused on the use of statistical and machine learning models in order to predict the future AQI values. McKeen [8] and Chuang [9] worked on real time air quality forecasting and developed online meteorological models to predict air quality. XU Xiaofeng [10] applied a method to determine that the type of pollution in Beijing is an ongoing process of research and found that low wind speed and air layer structural stability is the main cause of air pollution. Anikender Kumar [11] used the principal component regression technique in order to forecast the Air Quality Index (AQI) values in Delhi, India. Yongtao Hu [12] used synoptic classification for evaluating an operational air quality forecasting system in Atlanta. Computational models use a lot of the historical pollution data to predict the relationship between the input features and the output features by simple regression or complex machine learning methods. The absence of knowledge sources and the physical process do not significantly change the conditions of application of deterministic model [13].

There are a variety of machine learning algorithms in air pollution forecasting applications. Chen Chun Qi [14] applied multiple linear regression model in Wuhan to study the Wuhan Meteorological Environment impact and correlation on air quality. Pérez [15] obtained a model for forecasting PM_10_ values using the neural networks and compared it to the obtained linear model. Li [16] compared various artificial intelligence and machine learning models for air pollution forecasting. Bai Heming [17] used Neural Networks algorithm for forecasting the Air Quality Index. Shad [18] and Alhanafy [19] used the FC (Fuzzy logic) algorithm for predicting air pollution. Zolghadri [20] and Hoi [21] used the KF (Kalman filter) algorithm for predicting air quality parameters. Liu [22] used a Back-Propagation Neural Network and a Selection Sample Rule for forecasting Urban Air Quality. Li Xiang [23] also used GAB and fuzzy BP neural network for Air quality forecasting. Sun [24] used HMM (Hidden Markov Model) algorithm for prediction of PM_2.5_ concentrations in Northern California. Support Vector Regression [25] is a proven and wildly used machine learning algorithm for robust and reliable prediction results. It is also known for handling multi-dimensional data sets [26]. Also, SVR specializes for small number of samples for training [27].

This study is different from the past studies on air quality from start to the changes in input characteristic variables while observing the interaction of different conditions of urban air pollution. The present study examines the correlation between the same areas of urban air pollution and takes into account the air quality information of several cities together as input variables in order to predict the AQI using the Support Vector Regression (SVR) method with an aim to further improve the AQI forecast accuracy.

The harmful health effects of air pollution are because of multiple pollutants present in the atmosphere that cannot be justified with a single pollutant index. Hence a multiple pollutant AQI model is more effective than the presently utilized single pollutant model in modelling air quality across the pollutant concentrations. We have chosen the SVR algorithm for our study. The reason that SVMs often outperform Artificial Neural Networks (ANNs) in practice is that they deal with the biggest problem with ANNs: overfitting. SVR is less prone to overfitting than the ANNs due to the presence of regularization parameters. In SVR, the basic idea is to map the multi-variate data into higher-dimensional feature space via a nonlinear mapping with the help of a kernel trick and then perform regression in this space that avoids difficulties of using linear functions in the high dimensional feature space and the final optimization problem is transformed into dual convex quadratic programmes.

## Data and method

### Air quality data

**Fig 1:**
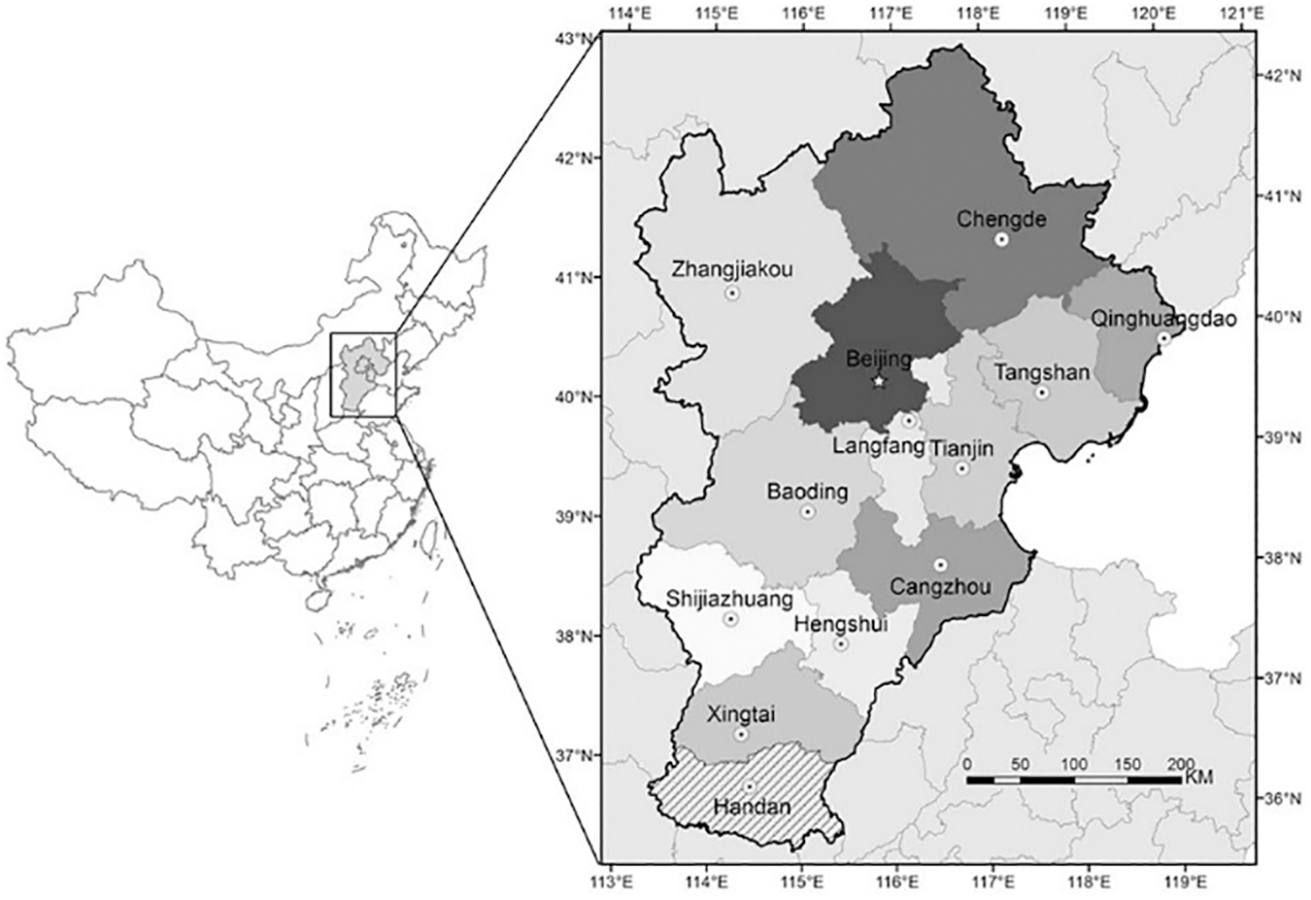
Jingjinji region. Map developed in ArcGIS (www.arcgis.com).

**Fig 2:**
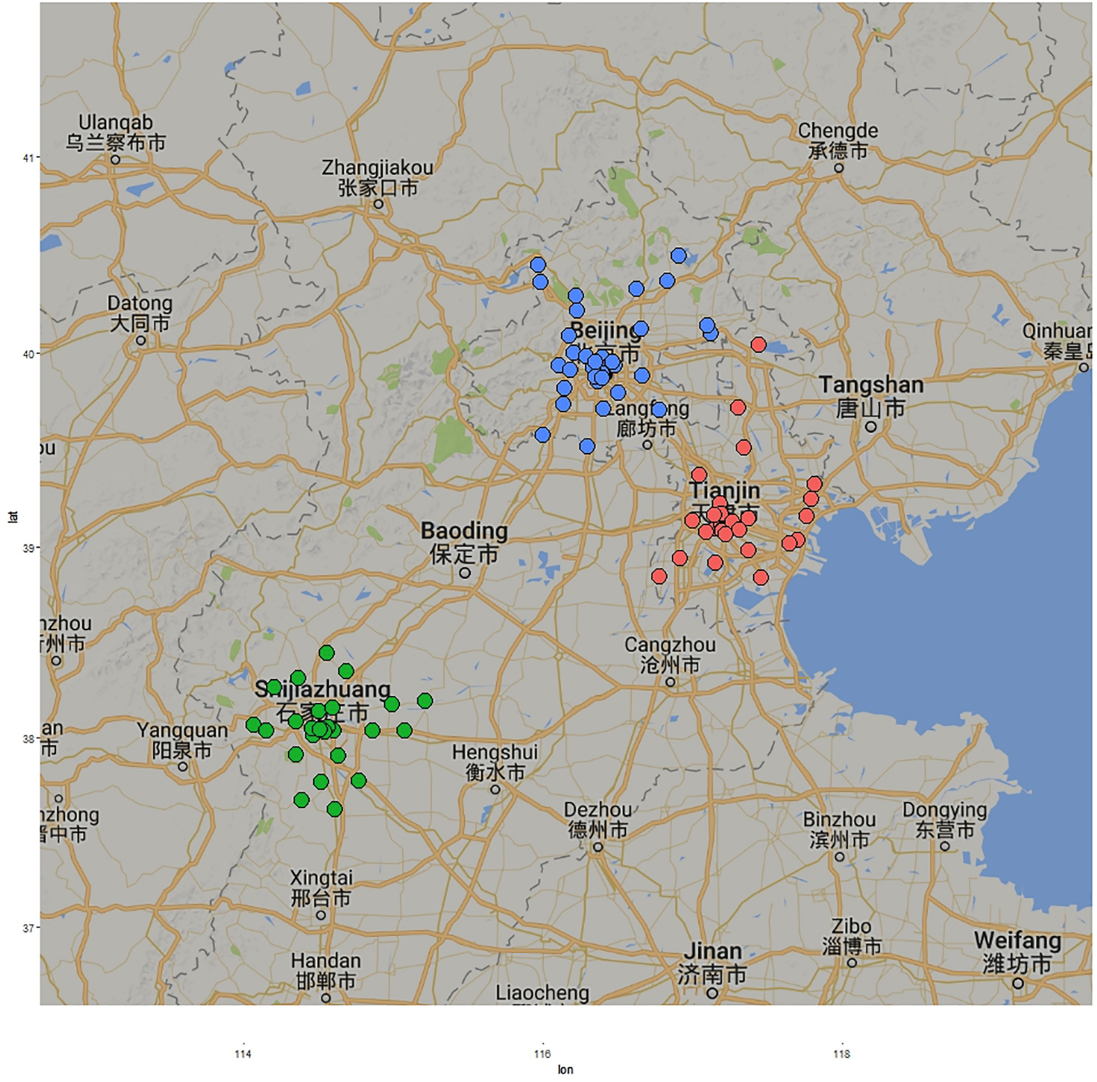
Map of air quality monitoring stations in Beijing (in blue), Tianjin (in red) and Shijiazhuang (in green). X-axis represents longitude and Y-axis represents latitude (Latitude and Longitude data is available in S1, S2 and S3 Tables). Generated using the ggplot2 (http://CRAN.R-project.org/package=ggplot2) and ggmap (https://cran.r-project.org/package=ggmap) packages of R.

Jingjinji represents the Beijing-Tianjin-Hebei region where Jing means Beijing, Jin means Tianjin and Ji means the Hebei region. According to geographical location characteristics of this capital economic area as well as the increase in urbanisation of Beijing-Tianjin-Hebei region (Fig 1), the paper selected Beijing, Tianjin and Shijiazhuang as the research objects. Fig 2 shows the exact location of the air quality monitoring stations that are used in our study. The pollutant concentrations and the AQI values for each city is the mean of the values obtained from the various monitoring stations around each city.

The data used consists of two parts: the daily air pollutant concentration data in three cities, obtained from the China Environmental Monitoring Center; the other part is the three cities daily weather condition and the meteorological data obtained from the China Meteorological Administration. The daily air pollutant concentration is calculated by selecting the maximum value from the eight-hour average concentration values. Hourly air pollutant concentrations are calculated using the eight-hour midpoint average concentrations i.e. for calculating the concentration for a particular hour, three consecutive next hours, four consecutive previous hours and that particular hour’s raw data is considered. Here we have considered a daily approach for Air Quality forecast primarily due to simplicity, practicality and operational reasons although the approach would have been similar if the hourly air pollutant data was considered. A total of 12 characteristic variables are considered for each city including the six air pollutants concentration namely "PM_2.5_", "PM_10_", "SO_2_", "CO", "NO_2_" and "O_3_"; five variable weather conditions namely "minimum temperature", " maximum temperature "," weather "," wind direction " and " wind power "; and the last day’s observed AQI values. The unit of measurement of air pollution features PM_2.5_, PM_10_, SO_2_, NO_2_ and O_3_ is μg/m^3^ and for CO is mg/m^3^. These are the most harmful pollutants present in large quantities in the atmosphere and therefore have a large amount of feature importance for AQI prediction. Hence the various automated air quality monitoring stations around each city focuses on only these six main pollutants mentioned above.

It is also important here to discuss about the various weather conditions included in our study for Air Quality forecast and modelling. Wind Direction sometimes has a substantial effect on the air quality of a particular city as well as a region. Air quality can either become better or worse based on wind direction. If the wind is coming from an area with extremely less pollution, then the air quality improves a significant amount. But if the wind is coming from a region that is highly polluted it is likely to become worse. Low wind speed for a highly polluted region with multiple sources of pollution is a problem because the pollution stays in the same region rather than blowing away in the direction of the wind [28]. Strong wind speeds generally promote the transport and travel of pollutants rapidly to distant places. High Temperature during the summer days contribute to photochemical reactions especially in the case of particulate matter and ozone. Whereas rain can clean the air but can cause problems of acid rain and soil pollution. In our study of the Jingjinji Region we have taken into account the features corresponding to these weather conditions. The feature weather is classified as partly cloudy, sunny, rainy, cloudy, snow, dust, haze and fog. These are represented by values 0 to 7 respectively shown in Table 1. Wind direction has been classified as north, northeast, east, south-east wind, southerly, north-west, west wind, south-west wind and no sustained wind nine types. These are represented by values 0 to 8 respectively as shown in Table 2. Wind Power has been summarized into 5 levels of wind power density based on the national standard of wind power in China: <3, 3–4, 4–5, 5–6 and 6–7 grade as shown in Table 3.

**Table 1:** Factors of input feature: Weather.

Weather	Factors
**Partly Cloudy**	0
**Sunny**	1
**Rainy**	2
**Cloudy**	3
**Snow**	4
**Dust**	5
**Haze**	6
**Fog**	7

**Table 2:** Factors of input feature: Wind direction.

Wind Direction	Factors
**North wind**	0
**North-east wind**	1
**east wind**	2
**South-east wind**	3
**south wind**	4
**North-west wind**	5
**west wind**	6
**South-west wind**	7
**No sustained wind**	8

**Table 3:** Factors of input feature: Wind power density.

Wind Power Levels	Wind Power Density (W/m^2^)	Factors
**<3 level**	<150	0
**3–4 level**	150–250	1
**4-5level**	250–300	2
**5–6 level**	300–400	3
**6–7 level**	400–1000	4

### Support Vector Regression (SVR)

Support Vector Machines (SVM) is a machine learning algorithm that constructs hyperplanes for separating different classes and is generally used for analyzing data that has a categorical output variable. Whereas in case of a continuous numeric output variable we use regression analysis in place of classification called Support vector regression (SVR). An SVR model is used to obtain an approximate function *g*(*x*) from a given complex sample data G={(xi,yi)}(Ni=1). The main idea is to first map the non-linearly separable data into a higher dimensional linearly separable feature space and then using this feature space for computation using linear programming [29].

$$f(x)=\sum_{i=1}^{D}w_{i}\phi_{i}(x)+b$$ (1)

In the Eq (1), *ϕ*_*i*_(*x*) is characterized by variables *b* and *w*_*i*_, and it can be estimated from the data. When the data is non-linearly separable, we need to map the data into richer feature space where the data is separable. The minimum function coefficients *w*_*i*_ can be obtained by:

$$R[w]=\frac{1}{N}\sum_{i=1}^{N}{\left|f(x_{i})-y_{i}\right|}_{\epsilon }+\lambda {\left||w|\right|}^{2}$$ (2)

In the Eq (2), *λ* is a standardization constant and function |*f*(*x*_*i*_)−*y*_*i*_|_ϵ_ can be defined as:

$${\left|f(x_{i})-y_{i}\right|}_{\epsilon }=\left\{ \begin{array}{l} |f(x)-y|-\epsilon ,\;|f(x_{i})-y_{i}|\geq \epsilon \\ \;0,\;other \end{array}\right.$$ (3)

The minimizing function can also be expressed in the following form [30]:

$$f(x,\alpha ,\alpha^{*})=\sum_{i=1}^{N}(\alpha_{i}-\alpha_{i}^{*})k(x_{i},x)+b$$ (4)

Simultaneously, $\alpha_{i}\alpha_{i}^{*}=0$, $\alpha_{i},\alpha_{i}^{*}\geq 0$, *i* = 1,.,*N*, the inner product kernel function can be expressed as

$$k(x,y)=\sum_{j=1}^{D}\phi_{j}(x)\phi_{j}(y)$$ (5)

The coefficients $\alpha_{i}\;and\;\alpha_{i}^{*}$ can be obtained by using the following equation:

$$R(\alpha_{i}^{*},\alpha_{i})=-\varepsilon \sum_{i=1}^{N}(\alpha_{i}^{*}{+\alpha }_{i})+\sum_{i=1}^{N}y_{i}(\alpha_{i}^{*}{-\alpha }_{i})-\frac{1}{2}\sum_{i,j=1}^{N}\left(\alpha_{i}^{*}{+\alpha }_{i})(\alpha_{i}^{*}{-\alpha }_{i})k(x_{i},x_{j}\right)$$ (6)

Constraint to $\sum_{i=1}^{N}(\alpha_{i}^{*}{-\alpha }_{i})=0,\;\alpha_{i}\geq 0,\;\alpha_{i}^{*}\leq C$.

For our present study we have used R programming language [31] for Support Vector Regression in order to forecast a continuous-valued attribute i.e. Air Quality Index (AQI) in our case.

### Multi-dimensional collaborative SVR model

**Fig 3:**
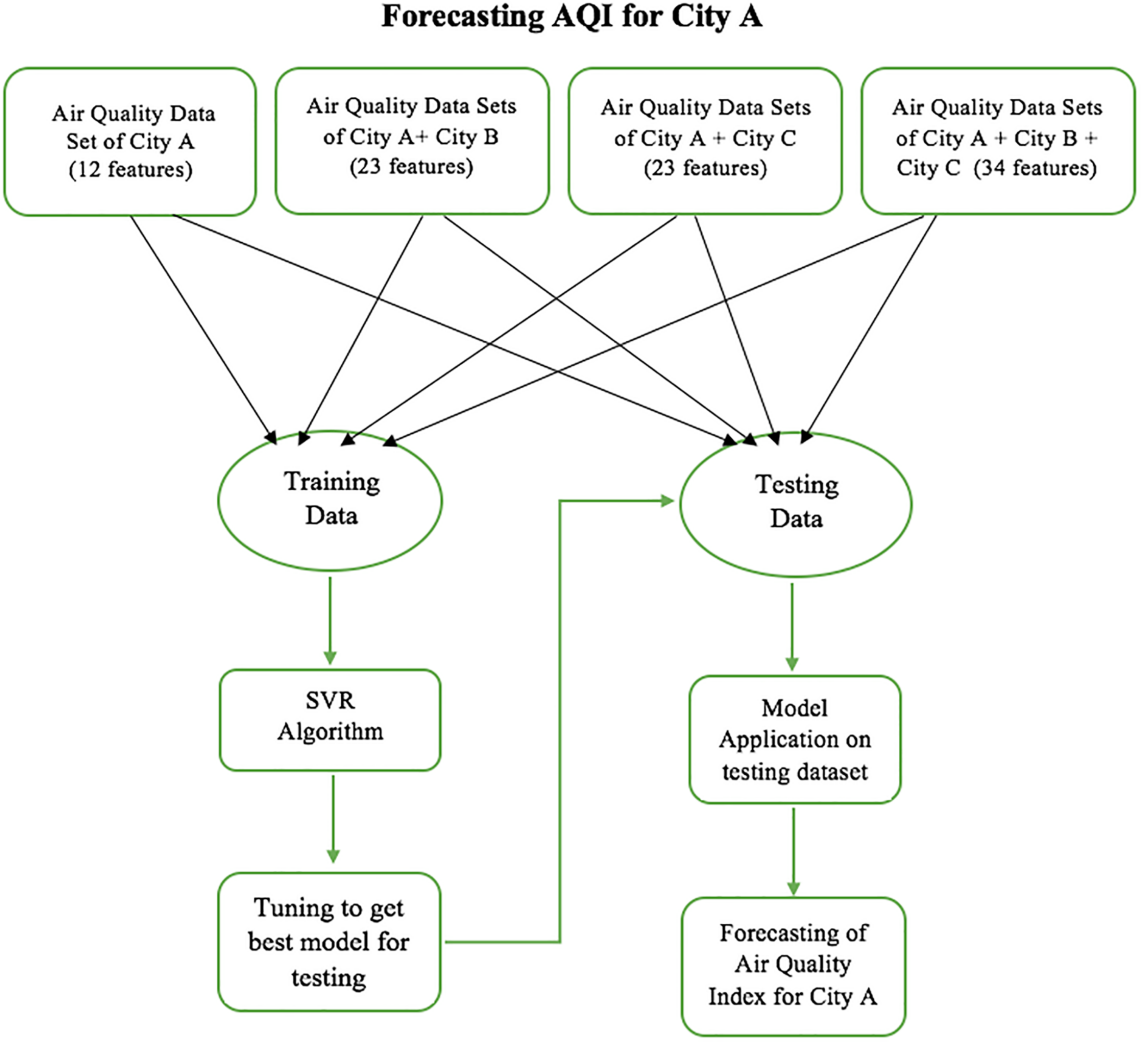
Multi-dimensional codimensional collaborative SVR Model for forecasting AQI values of City A using air quality data sets of city A, city A + city B, city A + city C, city A + city B + city C.

The aim of this study is to present a new model for AQI forecasting using collaborative multiple city air quality data as input. The structured flowchart representation of the proposed model is clearly shown below in Fig 3.

In order to forecast the Air Quality Index (AQI) values for city A, we take into account the air quality data of neighboring cities B and C along with city A. We take 3 cases—firstly we take air quality data of City A and City B as input; secondly we take air quality data of City A and City C as input and finally we take air quality data of all 3 cities as input in order to forecast the AQI values for city A. After this we split the three data sets into training and testing data. Then we develop SVR machine learning algorithm on all the three training data sets. Finally, Forecasting is carried out on the testing dataset based on the developed model on the training set. Since the number of input variables or features are increasing when we take into consideration the information from more than one city, the training complexity increases that leads to larger time to train the data. After the training the number of support vectors are selected and at the time of testing the complexity is linear on the number of the support vectors and linear on the number of features. This may vary for different kernels.

## Results and analysis

### Data distribution

Prediction and forecasting leads us into unknown territory. During the development of a predictive model we must assess its accuracy, reliability and credibility. Hence it is very important to divide the available data into separate partitions, developing our models on one of these partitions and using the other for predictive model assessment and validating it for possibly model refinement. The model development is done on the training set and the prediction is carried on the testing set. For the present study we used 4-fold cross validation technique in order to get accurate and credible results. We split the data into 4 folds with 25% data into each fold. Now we selected 3 folds for training the model and the remaining fold for testing the model. This was done until all the folds for training as well as testing were exhausted in order to avoid any bias in the dataset and also avoid any variations due to different seasons. A total of 851 days daily data is used for our study starting from January 1, 2014 to April 30, 2016. For each experiment the data division into 4 folds of training and testing datasets which is shown below in Table 4.

**Table 4:** 4-fold cross validation training and testing data division for Beijing, Tianjin and Shijiazhuang.

Sl. No.	Data (training/testing)	Duration	No. of data points (days)
1	Train 1	01.01.2014–30.09.2015	638
	Test1	01.10.2015–30.04.2016	213
2	Train 2	01.08.2014–30.04.2016	639
	Test 2	01.01.2014–31.07.2014	212
3	Train 3	02.03.2015–31.07.2014 *	638
	Test 3	01.08.2014–01.03.2015	213
4	Train 4	30.09.2015–01.03.2015**	639
	Test 4	02.03.2015–29.09.2015	212

* 02.03.2015–31.07.2014 represents data from 2^nd^ March 2015 to 30^th^ April 2016 and from 1^st^ January 2014 to 31^st^ July 2014.

** 30.09.2015–01.03.2015 represents data from 30^th^ September 2015 to 30^th^ April 2016 and from 1^st^ January 2014 to 1^st^ March 2015.

### Beijing-Tianjin-Shijiazhuang air quality index forecast

#### Beijing air quality index forecast

For Beijing AQI forecast, the experimental analysis is done taking into account the AQI information of different cities in 4 cases. In the first case, individual air pollutants and meteorological data of Beijing namely “PM_2.5_", "PM_10_", "SO_2_", "CO", "NO_2_" and "O_3_"; five variable weather conditions namely "minimum temperature", " maximum temperature "," weather "," wind direction " and " wind power " along with the last day’s observed AQI values are taken as 12 (11+1) input characteristics variables to predict Beijing’s AQI as an output variable using the SVR algorithm. In the second case, the individual air pollutants and meteorological data of Beijing as well as Shijiazhuang along with the last day’s observed AQI values are taken as 23 (11+11+1) input characteristics variables to predict Beijing’s AQI as an output variable. The third case is exactly similar to the 2^nd^ case except for the fact that we use Tianjin’s data instead of Shijiazhuang’s data. Finally, in the 4^th^ case we use the individual air pollutants and meteorological data of all the three cities–Beijing, Tianjin and Shijiazhuang along with the last day’s observed AQI values are taken as 34 (11+11+11+1) input characteristics variables to predict Beijing’s AQI as an output variable using SVR.

We have used 4-fold cross validation for estimating the error of our models. The 4-fold cross-validation estimator has a lower variance than a single set estimator. If we take a single set, where 75% of data are used for training and 25% used for testing, the test set is very small, hence there will be a lot of variation in the performance estimate for different samples or different partitions of the data to form training and test sets. 4-fold validation reduces this variance by averaging over 4 different partitions, so the performance estimate is less sensitive to the partitioning.

**Fig 4:**
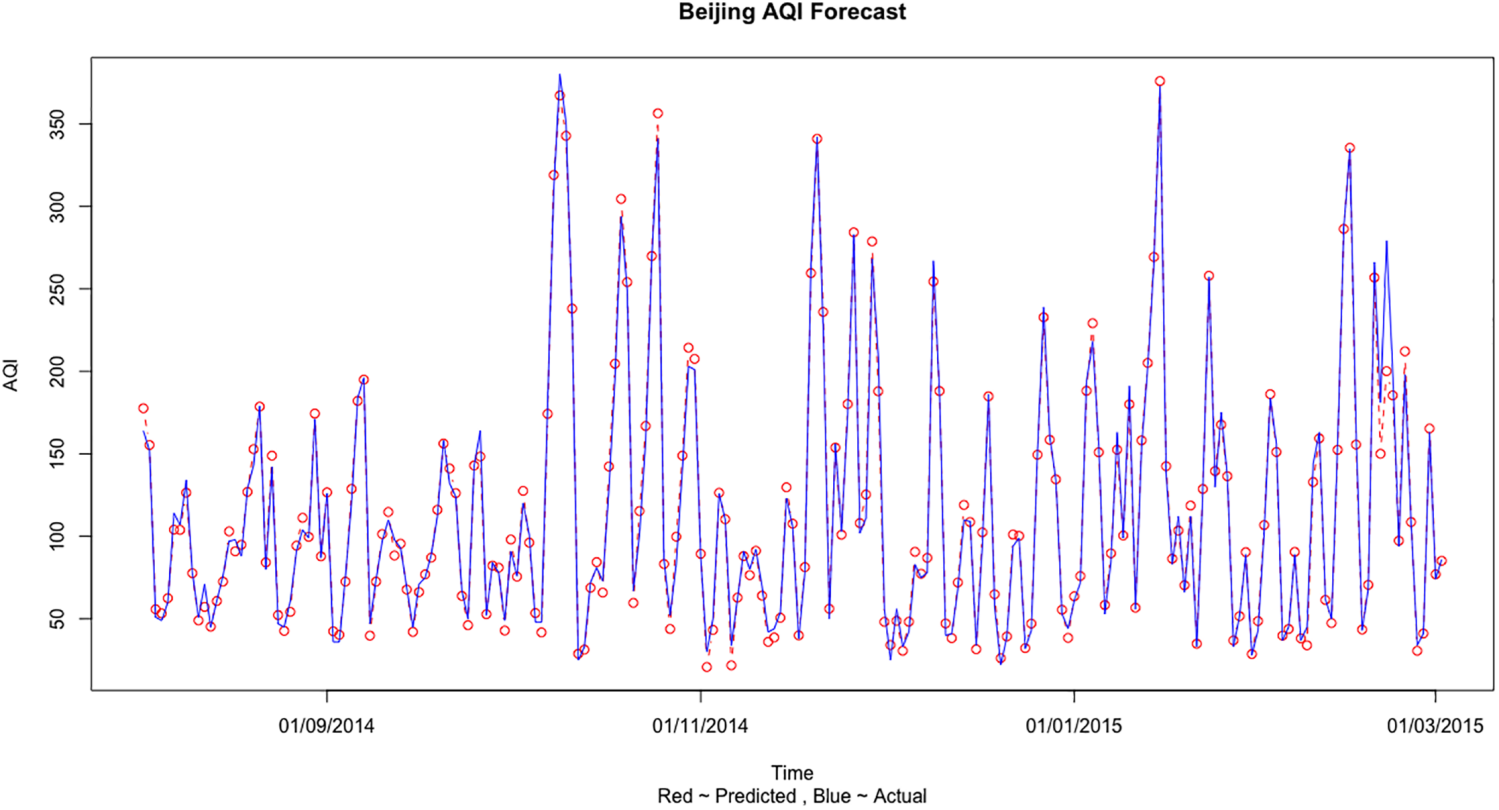
The comparison of actual and predict values for Beijing AQI forecast based on three city information for Test 3 dataset.

**Fig 5:**
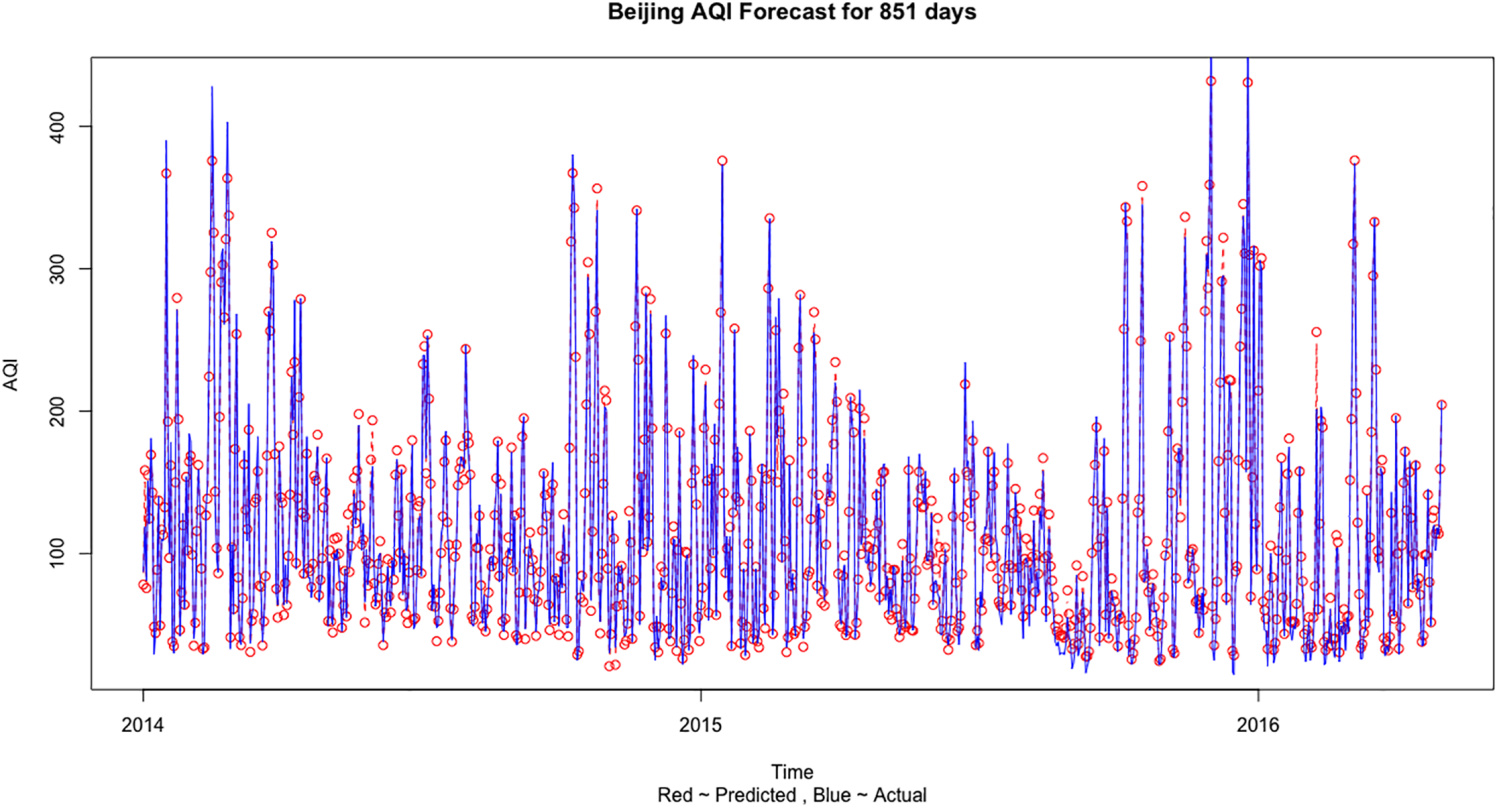
The comparison of actual and predict values for Beijing AQI forecast based on three city information for the complete dataset.

We select MSE (mean square error), the experimental prediction RMSE (root mean square error), MAE (mean absolute error) and MAPE (mean absolute percentage error) to calculate the prediction errors in all the 4 cases and finally to judge the performance of the different model. MSE, RMSE and MAE are scale dependent measures based on squared and absolute error values. MAE is generally smaller than the RMSE and is less sensitive to the large error values as it takes the absolute values and does not square the error value. But still MAE is a common and popular measure for comparing different methods on the same data set due to its simplicity and ease of calculation. On the other hand, MAPE is based on percentage error values and have the advantage of being scale independent. Hence it is the best measure to compare the forecast performance of the Support Vector Regression model between different datasets and needs to be minimum possible. The two most important measures are RMSE and MAPE. The RMSE values of the training and the testing datasets should be less than 12 and almost similar for the training and the testing datasets to conclude that the SVR model is strong and reliable. The MAPE for all the cases should falls between 0.05 ~ 0.09 to indicate accurate prediction results. The two significant digits for RMSE and four significant digits for MAPE are used in Table 5 to clearly show the difference in error values for different air quality information. From Table 5, the prediction error is minimized in the 4^th^ case when we use the Beijing, Tianjin and Shijiazhuang three cities information to predict the AQI values. Fig 4 shows the comparison of the predicted and actual values when training was done on Train 3 dataset and testing was done on Test 3 dataset for Beijing AQI forecast using all 3 city information. Fig 5 shows the comparison of actual and predict values for Beijing AQI forecast based on three city information for the complete dataset.

**Table 5:** Beijing AQI forecast results comparison based on different urban air quality information.

City Information	MSE	RMSE	MAE	MAPE
Beijing (1 city)	124.11	11.07	7.55	0.0914
Beijing + Shijiazhuang (2 City)	115.39	10.67	7.44	0.0911
Beijing, Tianjin (2 city)	107.20	10.33	7.42	0.0855
Beijing, Tianjin, Shijiazhuang (3 city)	87.69	9.35	6.66	0.0844

#### Tianjin and Shijiazhuang air quality index forecast

**Fig 6:**
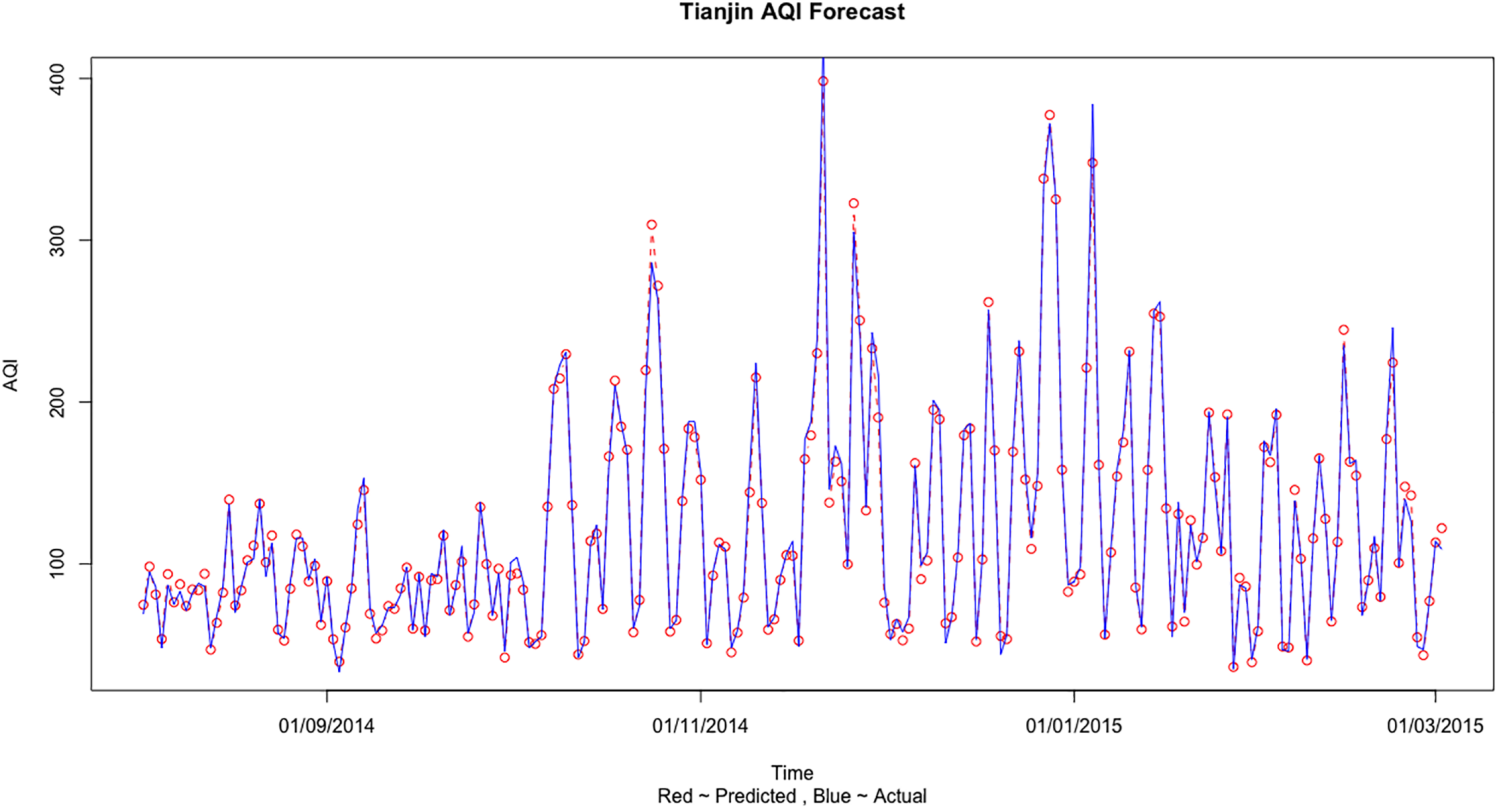
The comparison of actual and predicted values for Tianjin AQI forecast based on 3 city information for Test 3 dataset.

**Fig 7:**
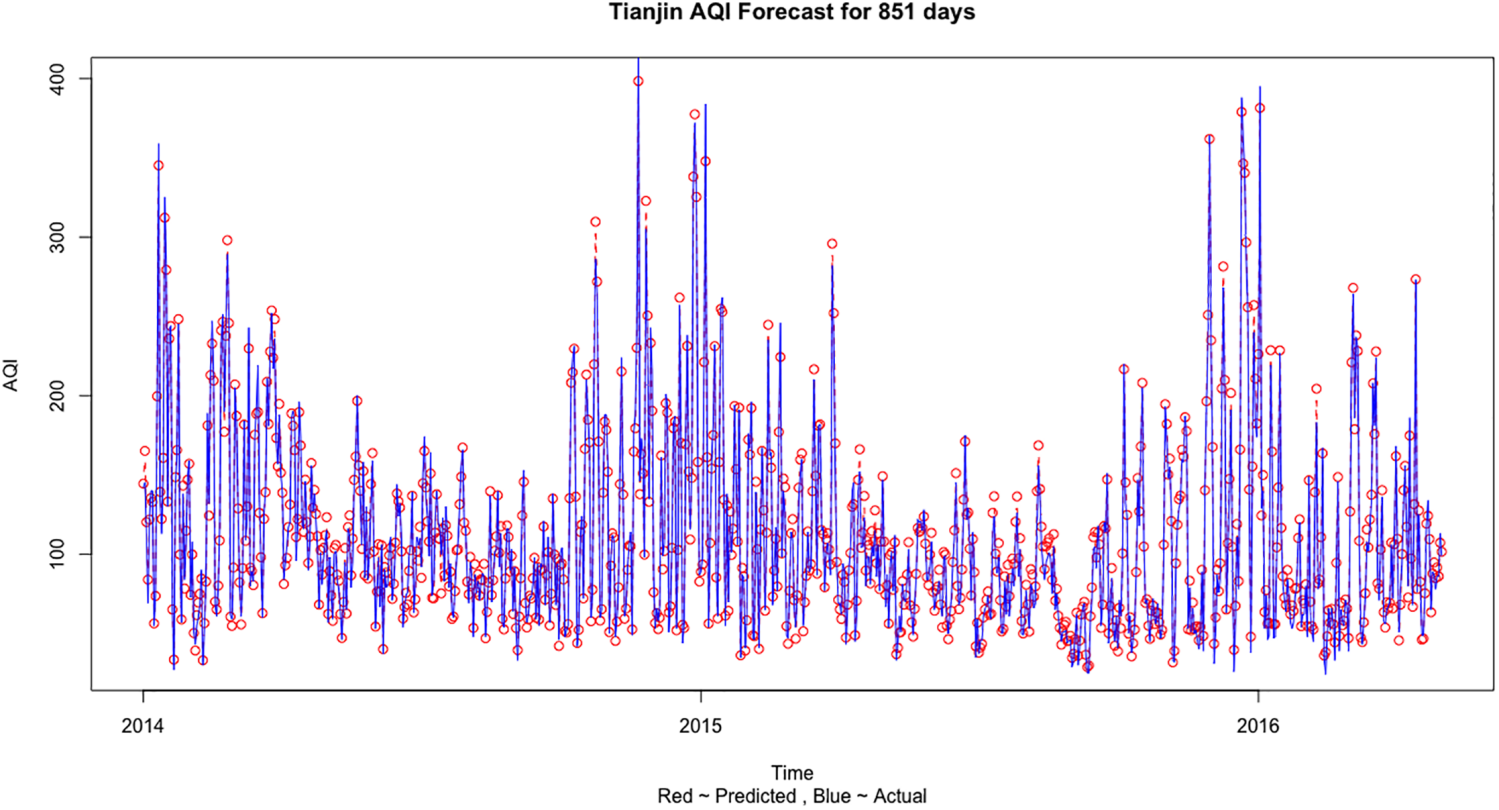
The comparison of actual and predict values for Tianjin AQI forecast based on three city information for the complete dataset.

Tianjin’s and Shijiazhuang’s AQI forecast is done in a similar way as described for Beijing above. Similar to Beijing, in Table 6, the prediction error is minimized in the fourth case when we use the Beijing, Tianjin and Shijiazhuang three cities information to predict the AQI values. Fig 6 shows the comparison of the predicted and actual values when training was done on Train 3 dataset and testing was done on Test 3 dataset for Tianjin AQI forecast using all 3 city information. Fig 7 shows the comparison of actual and predict values for Tianjin AQI forecast based on three city information for the complete dataset.

**Table 6:** Tianjin AQI forecast results comparison based on different urban air quality information.

City Information	MSE	RMSE	MAE	MAPE
Tianjin (1 city)	63.75	7.97	5.85	0.0597
Tianjin, Shijiazhuang (2 city)	61.01	7.78	5.75	0.0584
Tianjin, Beijing (2 city)	56.26	7.44	5.53	0.0579
Beijing, Tianjin, Shijiazhuang (3 city)	42.78	6.54	4.90	0.0534

**Fig 8:**
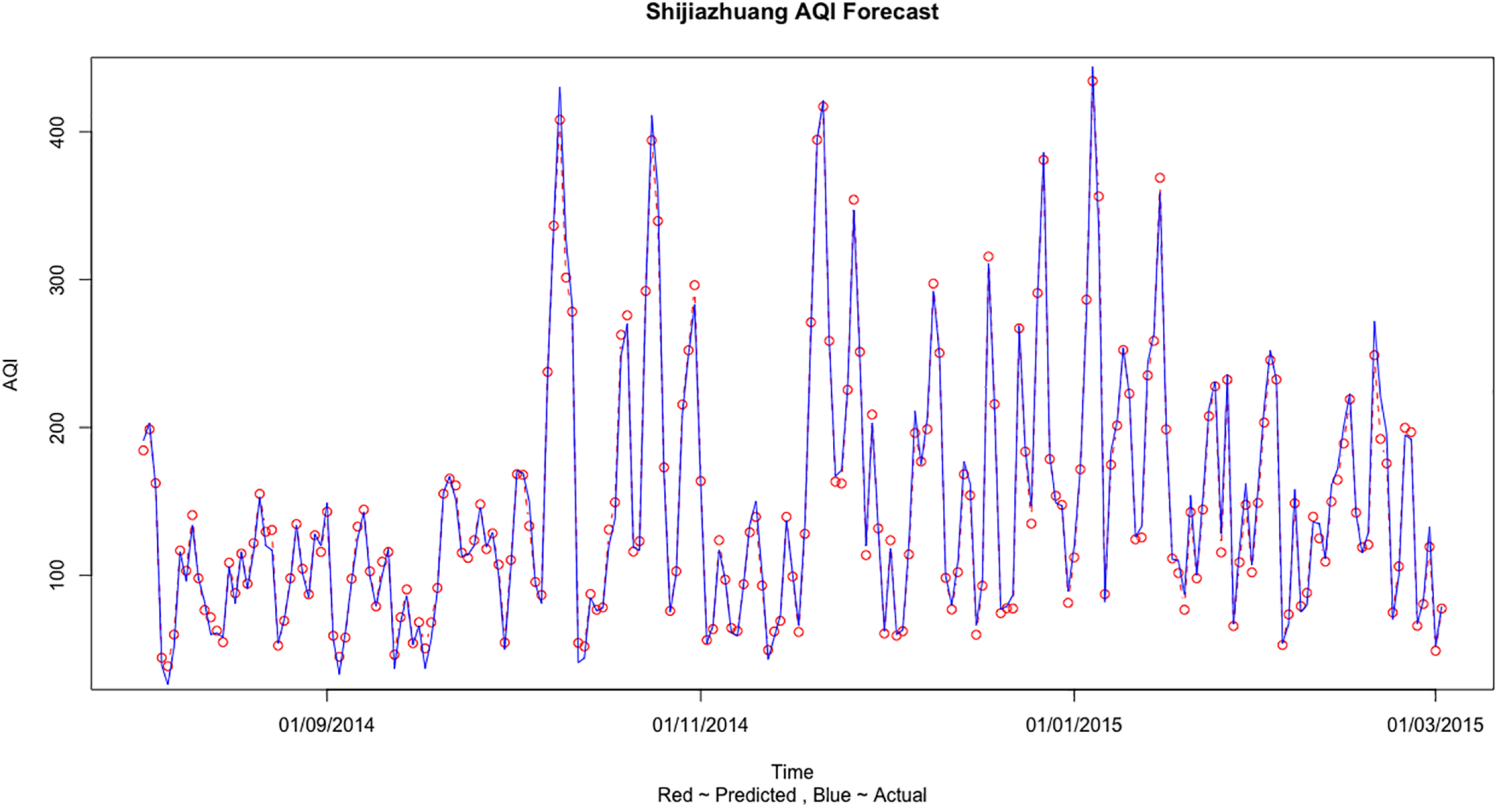
The comparison of actual and predicted values for Shijiazhuang AQI forecast based on its own city information for Test 3 dataset.

**Fig 9:**
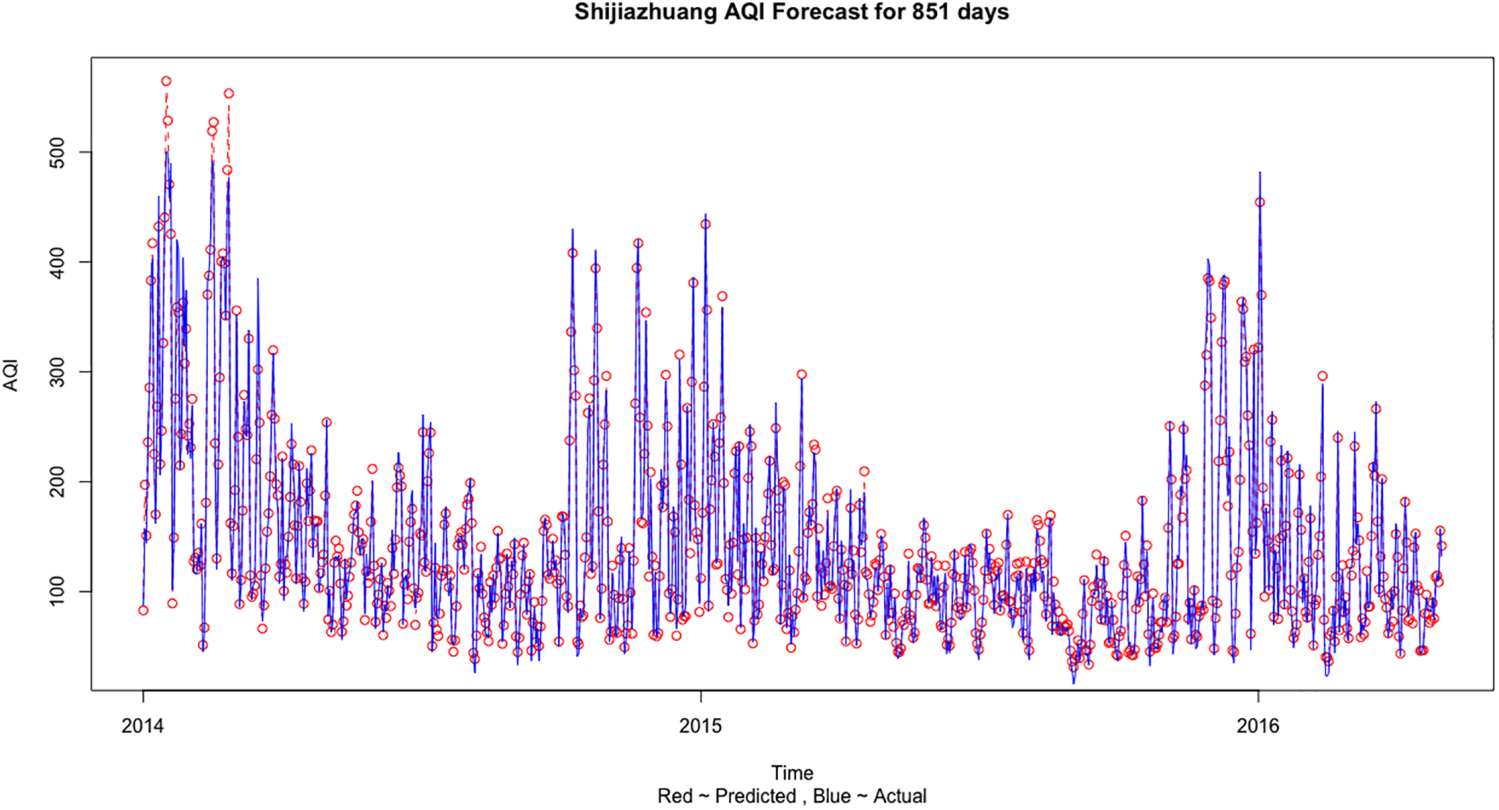
The comparison of actual and predict values for Shijiazhuang AQI forecast based on its own city information for the complete dataset.

In the case of Shijiazhuang, we see in Table 7 that the prediction error is minimized in the first case when we use only the Shijiazhuang information to predict the AQI values. Fig 8 shows the comparison of the predicted and actual values when training was done on Train 3 dataset and testing was done on Test 3 dataset for Shijiazhuang AQI forecast using only one city information. Fig 9 shows the comparison of actual and predict values for Shijiazhuang AQI forecast based on its own city information for the complete dataset.

**Table 7:** Shijiazhuang AQI forecast results comparison based on different urban air quality Information.

City Information	MSE	RMSE	MAE	MAPE
Shijiazhuang (1 city)	106.22	9.66	6.77	0.0590
Shijiazhuang, Tianjin (2 city)	112.82	10.25	7.29	0.0620
Shijiazhuang + Beijing (2 city)	122.76	10.70	7.48	0.0663
Beijing, Tianjin, Shijiazhuang (3 city)	128.70	11.01	7.72	0.0731

## Discussion

For our model, we have a single algorithm and we are working with different datasets for forecasting and establishing geographical relations. Hence we required a scale independent performance measure for comparing the same algorithm on various datasets. MAPE solves this purpose. From the three cities AQI forecast results it can be observed that surrounding cities air quality information helps in improving forecasting results when there is strong interaction and correlation between the input and the output features. It is also important to note the existence of differences in air quality interaction when we take multiple city air quality information as input. It can be observed that the in order to improve the prediction accuracy, Beijing’s AQI forecast needs the help of the air quality information of Tianjin and Shijiazhuang (Table 5). Table 5 clearly shows the reduction in prediction error (MAPE) for our proposed model compared to the single city air quality prediction. Similarly, in the case of Tianjin’s AQI forecast, Beijing, Shijiazhuang and Tianjin’s air quality information gave the best forecasting results (Table 6). Table 6 clearly shows the reduction in prediction error (MAPE) for the proposed model compared to the single city air quality prediction. Finally, in the case of Shijiazhuang AQI forecast, MAPE is minimum when we use only Shijiazhuang information to predict the AQI values (Table 7). Shijiazhuang air quality is not affected by air quality information of Beijing and Tianjin.

The results can be explained and some important conclusions can be made from the air pollution sources and geographical point of view. Beijing is a social services oriented political capital with multiple high-tech industries. Tianjin is an important economic center as well as an advanced manufacturing center in electronic information, aerospace and automobile manufacturing. Shijiazhuang is the leading heavy industry base based on steel, pharmaceutical, coal and chemical industries. Due to the different cities functional orientations, the sources and amount of air pollution are also different. It can be clearly seen that in general the AQI values observed in Shijiazhuang is more than Beijing and Tianjin (on comparing Figs 5, 7 and 9). Due to the more air pollution in Shijiazhuang, the surrounding areas of Tianjin and Beijing are also affected. So, Shijiazhuang air quality information will play an important role in Beijing, Tianjin and Shijiazhuang AQI forecast. It is important to look at the geographical locations of the three cities (see Fig 1). It can be seen that Beijing and Tianjin are located very close and they also share common boundaries. Therefore, we saw a similar forecasting result for both the cities. Whereas Shijiazhuang is a bit farther away from both Beijing and Tianjin and hence we see a different result for Shijiazhuang AQI forecast. Also, on comparing the 2-city and 3-city results for Beijing AQI forecast in Table 5, the decrease of MSE is more evident when Tianjin is introduced in the model (115.39 to 87.69) than Shijiazhuang (107.20 to 87.69). Thus it can be inferred from our observations that the geographical location plays an important role in AQI forecast.

In this paper we have used U.S. AQI based on daily values of several real time pollutant concentrations and the decision on using this indicator scheme was based on simplicity rather than on exact scientific reasoning. In order to further improve the air quality forecast in the future it is advisable to adopt the Air quality indicators that are close to the atmospheric reality [32]. These air quality indicators when used with hourly air pollutant concentration data could help in dealing with the auto-cancelling effects and rapid chemical transformation of some of the pollutants after they are released in the atmosphere.

## Conclusion

For the present study we selected different urban air quality information of Beijing, Tianjin and Shijiazhuang for AQI forecasting. By conducting the three cities AQI forecasting experiments we have reached to the following conclusions: The RMSE values of the training and the testing datasets are <12 and are almost similar for the training and the testing datasets for most of the cases, hence we can conclude that the support vector regression model is strong and reliable for predicting the AQI values. If the RMSE values for the test set is very higher than that of the training set then there is a problem of overfitting the data i.e. the model performs well on the training set but fails to give good predictions on the test set. Also the MAPE for all the cases falls between 0.05 ~ 0.09 and indicates highly accurate prediction result.The analysis shows that Shijiazhuang suffers a more serious air pollution because of its heavy industry base and needs adjustments and industrial upgrading to improve its present conditions. This new prediction method for air quality forecasting could provide better scientific basis for this kind of research work. This study was done on a particular region for a small period of time but it still shows that by taking careful advantage of the multiple city information based on the geographical location as well as using the weather conditions, we can expect a significant improvement in the forecasting results. We understand that in order to generalise the results elsewhere or for a different temporal series will require future readjustments and some modifications. This paper also opens the doors for further research and exploration of different forecasting methods and machine learning techniques in order to achieve better accuracy of air quality prediction in regression mode. Further research work could also be to use deep learning models along with multi city forecasting which can automatically extract the useful information required to forecast.

## Supporting information

S1 Table Latitude and longitude values of air quality monitoring stations in Beijing. (DOCX)

S2 Table Latitude and longitude values of air quality monitoring stations in Tianjin. (DOCX)

S3 Table Latitude and longitude values of air quality monitoring stations in Shijiazhuang. (DOCX)
