# Supplementary material for: Urban air quality forecasting based on multi-dimensional collaborative Support Vector Regression (SVR): A case study of Beijing-Tianjin-Shijiazhuang
Source: PLoS One. 2017 Jul 14;12(7):e0179763. doi: 10.1371/journal.pone.0179763 (PMC5510805; doi:10.1371/journal.pone.0179763)
Supplement: S1 Table — (DOCX) [file pone.0179763.s001.docx]

| **Latitude** | **Longitude** | **Beijing Station Name** |
| --- | --- | --- |
| 40.292 | 116.22 | Changping Dingling, Beijing |
| 40.217 | 116.23 | Changping Town, Beijing |
| 39.982 | 116.397 | Chaoyang Olympic Sports Center, Beijing |
| 39.937 | 116.461 | Chaoyang Agricultural Exhibition Hall, Beijing |
| 39.718 | 116.404 | Huangcunzhen, Daxing, Beijing |
| 39.929 | 116.417 | Dongcheng Dongsi, Beijing |
| 39.886 | 116.407 | Temple of Heaven, Dongcheng, Beijing |
| 39.939 | 116.483 | East Fourth Ring Road, Beijing |
| 39.742 | 116.136 | Fangshan, Liangxiang, Beijing |
| 39.824 | 116.146 | Fengtai Yungang, Beijing |
| 40.09 | 116.174 | The Haidian northern New Area, Beijing |
| 40.002 | 116.207 | Haidian Beijing Botanical Garden, Beijing |
| 39.987 | 116.287 | Haidian Wanliu, Beijing |
| 40.328 | 116.628 | Huairou town, Beijing |
| 40.499 | 116.911 | Miyun Reservoir, Beijing |
| 40.1 | 117.12 | Donggaocun Zhen, Pinggu, Beijing |
| 39.712 | 116.783 | Yongledianzhen, Tongzhou, Beijing |
| 39.52 | 116.3 | Yufazhen, Daxing, Beijing |
| 40.365 | 115.988 | Badaling Northwest, Beijing |
| 39.58 | 116 | Liulihezhen, Fangshan, Beijing |
| 39.937 | 116.106 | Mentougou, Longquan Town, Beijing |
| 40.37 | 116.832 | The town of Miyun, Beijing |
| 39.856 | 116.368 | South Ring Road, Beijing |
| 40.143 | 117.1 | Pinggu town, Beijing |
| 39.899 | 116.395 | Qianmen E St, Dongcheng, Beijing |
| 39.914 | 116.184 | Shijingshan city, Beijing |
| 40.127 | 116.655 | Shunyi New Town, Beijing |
| 39.886 | 116.663 | Tongzhou New Town, Beijing |
| 39.95459 | 116.4681 | Beijing US Embassy, Beijing |
| 39.929 | 116.339 | West Park officials, Beijing |
| 39.878 | 116.352 | West Wanshou Nishinomiya, Beijing |
| 39.954 | 116.349 | Xizhimen N St, Beijing |
| 40.453 | 115.972 | Yanqing town, Beijing |
| 39.795 | 116.506 | BDA, Beijing |
| 39.876 | 116.394 | Yongdingmen Inner St, Beijing |
