# Supplementary material for: Urban air quality forecasting based on multi-dimensional collaborative Support Vector Regression (SVR): A case study of Beijing-Tianjin-Shijiazhuang
Source: PLoS One. 2017 Jul 14;12(7):e0179763. doi: 10.1371/journal.pone.0179763 (PMC5510805; doi:10.1371/journal.pone.0179763)
Supplement: S2 Table — (DOCX) [file pone.0179763.s002.docx]

| **Latitude** | **Longitude** | **Tianjin Station Name** |
| --- | --- | --- |
| 39.51627 | 117.3431 | Baobai highway, Tianjin |
| 38.94317 | 116.9148 | Beiwei No.2 road, Tianjin |
| 39.11083 | 117.2452 | Dazhigu No.8 road, Tianjin |
| 39.03432 | 117.7072 | No.4 street, Tianjin |
| 40.04175 | 117.4444 | Donghuan road, Tianjin |
| 39.09703 | 117.1512 | Fukang road, Tianjin |
| 38.84517 | 116.7794 | Guang'an street, Tianjin |
| 39.32815 | 117.8166 | Guangming Road, Tianjin |
| 39.07847 | 117.0902 | Haitai fazhan No.2 road, Tianjin |
| 39.15875 | 117.7642 | Hanbei road, Tianjin |
| 39.15107 | 117.3748 | Hangtian road, Tianjin |
| 39.24735 | 117.7918 | Hexi Yijing road, Tianjin |
| 39.22605 | 117.185 | Huaihe street, Tianjin |
| 39.7188 | 117.3042 | Jianshe Road, Tianjin |
| 38.98458 | 117.3747 | Jingu Road, Tianjin |
| 39.11991 | 117.1888 | Nanjing Road, Tianjin |
| 39.17295 | 117.1934 | Nankou road, Tianjin |
| 39.0927 | 117.2017 | Qianjin street, Tianjin |
| 39.16543 | 117.1446 | Qinjian street, Tianjin |
| 39.37262 | 117.044 | Quanzhou South road, Tianjin |
| 39.02097 | 117.6503 | Tanggu Yingkou street, Tianjin |
| 38.91943 | 117.1566 | Tuanpowa, Tianjin |
| 39.08416 | 117.201 | Tianjin |
| 39.13375 | 117.2688 | Xiangshan street, Tianjin |
| 39.13767 | 117.0039 | Xinhua street, Tianjin |
| 38.83945 | 117.4574 | Yongming road, Tianjin |
| 39.08714 | 117.3145 | Yuejin Road, Tianjin |
| 39.0635 | 117.225 | Zhujiang street, Tianjin |
