# Supplementary material for: Urban air quality forecasting based on multi-dimensional collaborative Support Vector Regression (SVR): A case study of Beijing-Tianjin-Shijiazhuang
Source: PLoS One. 2017 Jul 14;12(7):e0179763. doi: 10.1371/journal.pone.0179763 (PMC5510805; doi:10.1371/journal.pone.0179763)
Supplement: S3 Table — (DOCX) [file pone.0179763.s003.docx]

| **Latitude** | **Longitude** | **Shijiazhuang Station Name** |
| --- | --- | --- |
| 37.91 | 114.354 | Fenglong Montain, Shijiazhuang |
| 38.039 | 114.862 | Gaocheng Experimental School, Shijiazhuang |
| 38.04 | 114.605 | High-tech zone, Shijiazhuang |
| 37.623 | 114.607 | Gaoyi County City Hall, Shijiazhuang |
| 38.055 | 114.564 | Chemical College, Shijiazhuang |
| 38.071 | 114.069 | Jingxing kuangqu qu wei dalou, Shijiazhuang |
| 38.038 | 114.152 | Jingxing Bureau of Meteorology, Shijiazhuang |
| 38.04 | 115.079 | Jin zhou bo na de, Shijiazhuang |
| 38.315 | 114.367 | Ling shou gongshui, Shijiazhuang |
| 37.906 | 114.633 | Luancheng Communications, Shijiazhuang |
| 38.085 | 114.347 | Lu quan yi zhong, Shijiazhuang |
| 38.266 | 114.204 | Pingshan Ye River, Shijiazhuang |
| 38.052 | 114.521 | People's Hall, Shijiazhuang |
| 38.197 | 115.213 | Shenze Power Supply Bureau, Shijiazhuang |
| 38.031 | 114.542 | Shiji Park, Shijiazhuang |
| 38.178 | 114.991 | Wuji EPA, Shijiazhuang |
| 38.14 | 114.502 | Northwest Water Company, Shijiazhuang |
| 38.012 | 114.467 | Xinan College, Shijiazhuang |
| 38.444 | 114.56 | Xing tang xianwei bangong lou, Shijiazhuang |
| 38.349 | 114.69 | Xin yue shiwei dong lou, Shijiazhuang |
| 37.766 | 114.517 | Yuan shi zhu jian ju, Shijiazhuang |
| 37.672 | 114.392 | Zanhuang County City Hall, Shijiazhuang |
| 37.771 | 114.77 | Zhaoxian EPA, Shijiazhuang |
| 38.159 | 114.593 | Zhengding Unicom, Shijiazhuang |
| 38.051 | 114.455 | Worker hospital, Shijiazhuang |
| 38.04231 | 114.5149 | Shijiazhuang |
